# Medioresinol from Eucommiae cortex improves myocardial infarction-induced heart failure through activation of the PI3K/AKT/mTOR pathway: A network analysis and experimental study

**DOI:** 10.1371/journal.pone.0311143

**Published:** 2024-09-27

**Authors:** Xueting Qin, Xuan Liu, Can Guo, Li Huang, Qiyao Xu

**Affiliations:** 1 Nephrology, The Second People’s Hospital of China Three Gorges University, Yichang, Hubei, China; 2 Graduate School, Nanjing University of Chinese Medicine, Nanjing, Jiangsu, China; The Second Affiliated Hospital of Guangzhou Medical University, CHINA

## Abstract

**Objective:**

This study aims to systematically analyze the potential active components of Eucommiae cortex in the treatment of post- myocardial infarction heart failure through network analysis and molecular docking methods. In vitro experiments were conducted to verify that medioresinol, a component of Eucommiae cortex, improves oxygen-glucose deprivation-induced cell failure through its anti-inflammatory and antioxidant capacities.

**Methods:**

Potential active components of Eucommiae cortex were screened using specific data. The targets of these components were predicted using Swiss Institute of Bioinformatics database and TargetNet, and key targets were identified by intersecting with the disease targets of myocardial infarction and heart failure. Protein-Protein Interaction analysis was performed on the key targets to screen for core targets. Genomics Institute of the Novartis Research Foundation and Human Protein Atlas were used to identify myocardial highly expressed targets. Kyoto Encyclopedia of Genes and Genomes and Gene Ontology enrichment analyses were conducted using the Database for Annotation, Visualization, and Integrated Discovery. Molecular docking was performed for the final components and target proteins. In vitro experiments were carried out using H9c2 cells subjected to oxygen and glucose deprivation conditions to validate the effects of the screened potential active components.

**Results:**

Network analysis revealed that Eucommiae cortex might exert its effects through the phosphoinositide 3-kinase/protein kinase B/mammalian target of rapamycin (PI3K/AKT/mTOR), hypoxia-inducible factor 1, and Janus kinase/signal transducer and activator of transcription pathways, which are crucial for myocardial contraction, vascular tone regulation, inflammatory response, and oxidative stress. Molecular docking indicated stable binding of the selected compounds to PI3K, AKT, and mTOR. Medioresinol was selected for further study and shown to significantly improve oxidative stress and inflammatory response in myocardial ischemia-hypoxia model cells by activating the PI3K/AKT/mTOR pathway.

**Conclusion:**

This study confirms the role of the PI3K/AKT/mTOR pathway in the cardiovascular protective effects of Eucommiae cortex and provides evidence at the cellular level. Medioresinol demonstrated potential therapeutic effects on myocardial infarction induced heart failure by reducing oxidative stress and inflammatory responses. These findings offer a theoretical basis for the application of Eucommiae cortex in the treatment of heart failure and support the development of new therapeutic drugs for cardiovascular diseases. Future research should further validate these effects in animal models and explore the overall efficacy of Eucommiae cortex.

## Introduction

Heart failure (HF) represents the terminal stage of cardiovascular diseases, characterized by a complex pathogenesis involving various factors such as myocardial injury, neuroendocrine activation, inflammatory responses, and oxidative stress [1, 2]. The primary underlying causes of HF include hypertension, diabetes, and coronary artery disease, with myocardial infarction (MI) being a significant contributor to HF among coronary artery disease patients [3, 4]. Post-MI HF (post-MI HF) has a relatively high incidence. Although the risk of HF after MI has significantly decreased with the widespread use of reperfusion therapy, approximately 10% of ST-elevation MI patients still develop HF within the following years [5, 6]. The main reasons for HF after MI include myocardial cell necrosis and fibrosis [7]. On one hand, the reduction and loss of function of local myocardium post-MI can lead to acute HF; on the other hand, short-term inflammatory responses and long-term myocardial remodeling after MI can result in chronic HF. The treatment of post-MI HF does not significantly differ from that of general HF, primarily relying on pharmacotherapy and lifestyle modifications. Commonly used medications include beta-blockers, angiotensin receptor-neprilysin inhibitors, angiotensin-converting enzyme inhibitors, angiotensin II receptor blockers, and aldosterone receptor antagonists, which can inhibit myocardial remodeling, improve cardiac function, and enhance prognosis through various mechanisms [8]. Despite significant advancements in the treatment of acute MI, post-MI HF remains difficult to fully treat, with poor prognosis. Even though many current medications benefit HF patients, the all-cause mortality rate has not further declined [9]. Therefore, there is an urgent need to explore new therapeutic strategies to improve the long-term prognosis of patients with post-MI HF or to further prevent the occurrence of HF after MI.

Traditional Chinese medicine has unique advantages in treating cardiovascular diseases due to its multi-component, multi-target, and multi-pathway therapeutic effects [10]. Eucommiae cortex, a traditional Chinese botanical drug, contains major chemical constituents such as flavonoids, lignans, and phenolic acids, which possess a wide range of pharmacological activities [11, 12]. In traditional Chinese medicine, Eucommiae cortex is known for its properties of tonifying the liver and kidneys, strengthening bones and muscles, and preventing miscarriage. It is commonly used in the cardiovascular field to treat hypertension. Its antihypertensive effects were confirmed approximately half a century ago and are primarily attributed to its active component, pinoresinol diglucoside [13]. Modern pharmacological research has demonstrated that Eucommiae cortex also exhibits anti-inflammatory, antioxidant, anti-fibrotic, and immunomodulatory effects [14–17]. Since oxidative stress, inflammatory responses, and myocardial fibrosis are the main causes of HF following MI, Eucommiae cortex may have potential therapeutic value in the treatment of post-MI HF.

Although the pharmacological effects of Eucommiae cortex have been widely studied, its specific mechanisms in the treatment of HF remain unclear and under-researched, with studies focusing on post-MI HF being nonexistent. However, recent research suggests that the component of Eucommiae cortex, Aucubin, can effectively treat pressure overload-induced HF, primarily by activating the β(3)-adrenergic receptor/adenylyl cyclase/cyclic adenosine monophosphate pathway [18]. Due to the multi-component nature of traditional Chinese medicine, its mechanisms of action are relatively complex. To fully understand the therapeutic potential of Eucommiae cortex, it is necessary to adopt systematic research approaches to elucidate its mechanisms of action.

This study aims to systematically analyze the potential active components of Eucommiae cortex in the treatment of post-MI HF, as well as their potential targets and mechanisms, using network analysis and molecular docking methods. Furthermore, in vitro experiments will be conducted to verify the anti-inflammatory and antioxidant capacities of the screened potential active components in the treatment of MI-induced HF. It is hoped that this research will provide a theoretical basis for the application of Eucommiae cortex in the treatment of HF and offer scientific support for the development of new therapeutic drugs for HF. By systematically analyzing the multi-target and multi-pathway mechanisms of Eucommiae cortex, this study will help elucidate the holistic pharmacological action patterns of traditional Chinese medicine formulas, providing a reference for the modernization and internationalization of traditional Chinese medicine.

## Materials and methods

### Screening of effective components from Eucommiae cortex

Using the Traditional Chinese Medicine Systems Pharmacology Database and Analysis Platform (TCMSP, https://tcmsp.91medicine.cn/#/home), search for Eucommiae cortex, select “*ingredients*”, and download the entire dataset. Filter effective components based on oral bioavailability (OB) ≥ 30% [19], drug-likeness (DL) ≥ 0.18 [20], and molecular weight (MW) < 500 [21].

### Prediction of target proteins for effective components

The selected effective components were searched in PubChem to obtain their Simplified Molecular Input Line Entry System (SMILES). For those components without available SMILES, the molecular structure files (.mol2) provided by TCMSP were converted to SMILES using the Open Babel open-source tool (version 3.1.0, http://openbabel.org/). Subsequently, the potential targets were predicted using the Swiss Institute of Bioinformatics (SWISS) online tool (http://www.swisstargetprediction.ch/), selecting *Homo sapiens* and using the obtained SMILES. For molecules without predicted targets from the SWISS online tool, TargetNet (http://targetnet.scbdd.com/calcnet/index/) was used to predict the targets, with the settings kept at default parameters (Include models with AUC ≥0.7, Fingerprint type: ECFP4 fingerprints). The obtained data retained the target’s Uniprot ID and Probability information.

### Acquisition of disease targets

The targets for diseases were obtained using the DisGeNET database (https://www.disgenet.org/search). Specifically, searches were conducted for heart failure (CUI: C0018801) and myocardial infarction (CUI: C0027051). By clicking on "Summary of Gene-Disease Associations," the resulting data were saved, retaining the information on *Gene*, *Full name*, and *Uniprot ID*.

### PPI and key targets

The targets obtained in sections “prediction of target proteins for effective components” and “acquisition of disease targets” were intersected based on their Uniprot IDs to identify shared targets. The shared targets were then uploaded in bulk to the String database (https://string-db.org/) using the "Multiple proteins" function in the Search section, with the organism set to Homo sapiens. The resulting protein-protein interaction (PPI) data were directly imported into Cytoscape (version 3.10.2, https://cytoscape.org/). Using the Molecular Complex Detection plugin (degree cutoff: 2, k-core: 2), clusters with a node count of 4 or more were selected as core clusters. Subsequently, the Cytohubba plugin was used to further screen the targets in the core clusters, selecting the top 15 targets based on the Degree score and the top 15 targets based on the maximal clique centrality (MCC) score. The intersection of these two target groups was then taken to identify the key targets.

### KEGG pathway enrichment analysis and GO functional enrichment analysis

The targets obtained in sections “prediction of target proteins for effective components” and “acquisition of disease targets” were intersected based on their Uniprot IDs to identify shared targets. The Uniprot IDs of these shared targets were then uploaded in bulk to the Database for Annotation, Visualization, and Integrated Discovery (DAVID) (https://david.ncifcrf.gov/tools.jsp) for analysis. Data were extracted for the following four categories: Kyoto Encyclopedia of Genes and Genomes (KEGG) Pathway and Gene Ontology biological process (GOBP) terms Direct, GO cellular component (GOCC) terms Direct, and GO molecular function (GOMF) terms Direct. The results were saved as separate Excel files. For each table, entries with p-values, Bonferroni values, Benjamini values, and FDR values all less than 0.05 were selected. If the final data count exceeded 20, the 20 entries with the smallest p-values were retained. The data were then visualized in Python (version 3.11.7, https://www.python.org/) using pandas (version 2.2.2, https://pandas.pydata.org/), matplotlib (version 3.8.0, https://matplotlib.org/), and numpy (version 1.26.4,).

### Screening for highly expressed myocardial target proteins

Using the same method as described in section “KEGG pathway enrichment analysis and GO functional enrichment analysis”, the shared targets were imported into the DAVID database to extract Genomics Institute of the Novartis Research Foundation and Human Protein Atlas data. The proteins expressed in myocardial tissue were then filtered and selected.

### Screening for highly enriched KEGG and GO in myocardium

The target proteins obtained in section “screening for highly expressed myocardial target proteins” were merged based on their Uniprot IDs. The final list of highly expressed myocardial proteins was then imported into the DAVID database to extract and visualize KEGG and GO data using the method described in section “KEGG pathway enrichment analysis and GO functional enrichment analysis”.

### Construction of compound-target-KEGG/GO-disease network

The key proteins obtained in section “screening for highly expressed myocardial target proteins”, the key KEGG and GO terms screened in section “screening for highly enriched KEGG and GO in myocardium”, and the effective components of Eucommia targeting these key proteins were matched with the diseases to construct the compound-target-KEGG/GO-disease network. The network was visualized using Gephi software (version 0.10.1, https://gephi.org/).

### Molecular docking

Molecular docking was performed for the proteins and effective components of Eucommia mentioned in section “construction of compound-target-KEGG/GO-disease network”. The 3D structure files of the effective components were obtained from PubChem. If 3D structure files were not available, 2D structure files provided by TCMSP were converted to 3D structure files using Open Babel. Target proteins were searched on the Uniprot website using their Uniprot IDs. The structural data were reviewed to select the highest resolution single-chain structures, and the corresponding structure files were obtained from the RCSB Protein Data Bank (https://www.rcsb.org/).

All files were converted to.pdbqt format using AutoDockTools (ADT, version 1.5.7, https://autodock.scripps.edu/resources/adt/), and during this process, small molecule ligands, unnecessary metal ions, and other atoms were removed from the protein structure files. Molecular docking was then conducted using AutoDock Vina (version 1.1.2, https://vina.scripps.edu/), with the exhaustiveness set to 16 and other parameters kept at default settings. The conformations with the lowest binding energies from the docking results were visualized using PyMOL open-source version (version 2.6.0a0, https://pymol.org/2/).

### Cell culture and cell model establishment

The H9c2 cells used in this study were obtained from Procell (Catalog No. CL-0089, China). Routine culture was performed in a medium composed of 89% Dulbecco’s Modified Eagle Medium (with NaHCO_3_ 1.5g/L) (iCell-128-001, Cellverse, China), 10% fetal bovine serum (10270–106, Gibco, Thermo Fisher Scientific, USA), and 1% penicillin/streptomycin (C0222, Beyotime, China), in an incubator at 37°C with 5% CO_2_.

The oxygen and glucose deprivation (OGD) model was established following previous studies [22, 23]. Specifically, the medium was replaced with glucose-free medium, and the cells were placed in an incubator with 95% N_2_ and 5% CO_2_ (37°C) to create the ischemia-hypoxia model, simulating MI. After 6 hours, the cells showed reduced function, simulating HF. The glucose-free medium consisted of 89% glucose-free Dulbecco’s Modified Eagle Medium (iCell-138-0001, Cellverse, China), 10% fetal bovine serum, and 1% penicillin/streptomycin.

### Cell grouping and intervention

The final concentrations of the intervention compounds in the culture medium were as follows: 740 Y-P (CAS: 1236188-16-1, MCE, USA) at 12μM [24–26], Wortmannin (WTM) at 100 nM [27, 28]. The low dose of medioresinol (MDRN) (CAS: 40957-99-1, MCE, USA) is 60 μM, denoted as MDRN-L, and the high dose is 120 μM, denoted as MDRN-H. Cells were cultured under normal conditions for 48 hours, followed by 18 hours of drug intervention, and then subjected to 6 hours of OGD treatment based on the drug intervention. The cells were divided into seven groups: the control group, the OGD model group, the OGD + 740 Y-P group (positive drug group), the OGD + WTM group, the OGD + MDRN-L group, the OGD + MDRN-H group, and the OGD + MDRN-H + WTM group.

### Cell viability assay

Cell viability was measured using a commercially available CCK-8 assay kit (C0038, Beyotime, China) according to the manufacturer’s instructions. This assay was used to evaluate the final drug concentrations and the extent of HF.

### ELISA assays for NT-proBNP, TNF-α, and IL-1β

ELISA assays were conducted to measure N-terminal pro b-type natriuretic peptide (NT-proBNP), TNF-α, and IL-1β levels using commercially available kits: Rat NT-proBNP (20240408, DreamBio, China), Rat tumor necrosis factor-alpha (TNF-α) (20240427, DreamBio, China), and Rat interleukin-1 beta (IL-1β) (20240423, DreamBio, China). According to the manufacturer’s instructions, NT-proBNP was measured using cell lysates, while TNF-α and IL-1β were measured using the culture supernatant.

### Reactive oxygen species assay

Reactive oxygen species (ROS) levels were measured using a commercially available DCFH-DA ROS Detection Kit (S0033S, Beyotime, China). Specifically, after culturing the cells in a 96-well plate under OGD conditions for 4 hours, the detection reagent was quickly added, and the cells were further incubated under OGD conditions for another 2 hours. At the end of the incubation, ROS levels were directly measured using a Synergy H1 microplate reader (BioTek, USA) with excitation at 488 nm and emission at 525 nm.

### Mitochondrial membrane potential detection

Mitochondrial membrane potential was measured using a commercially available JC-1 Mitochondrial Membrane Potential Detection Kit (C2006, Beyotime, China). Cells were cultured in a 24-well plate, and after the culture period, the assay was performed according to the manufacturer’s instructions. Images were captured using a Vert A1 inverted fluorescence microscope (Zeiss, Germany), and ImageJ software (version 1.54f) was used to analyze the average fluorescence intensity of the red and green fluorescence. Specifically, the integrated density of red fluorescence (R) was measured in the red channel, and the integrated density of green fluorescence (G) was measured in the green channel. The mitochondrial membrane potential depolarization was expressed as the ratio of green fluorescence intensity to the sum of red and green fluorescence intensities: *G*/(*R*+*G*).

### Western blot

Western blotting was performed according to standard protocols. The primary antibodies used were phosphatidylinositol 3-Kinase (PI3K) p110 alpha Monoclonal antibody (67071-1-Ig, Proteintech, China), Phospho- protein kinase B (AKT) Monoclonal antibody (66444-1-Ig, Proteintech, China), Phospho- mammalian target of rapamycin (mTOR) Monoclonal antibody (67778-1-Ig, Proteintech, China), and Alpha Tubulin Monoclonal antibody (66031-1-Ig, Proteintech, China). The dilution ratios were 1:1000, 1:2000, 1:2000, and 1:25000, respectively. A total of 40 μg of protein was loaded per sample.

The transfer was performed using a semi-dry transfer system. The membrane for phosphorylated mTOR (p-mTOR) and Tubulin was from a 6% gel, transferred at 25V for 40 minutes. The membrane for PI3Kα, phosphorylated AKT (p-AKT), and Tubulin was from a 10% gel, transferred at 25V for 15 minutes.

Imaging was performed using the Azure Sapphire RGBNIR imaging system, set to medium exposure intensity with automatic exposure. Band intensity was analyzed using ImageJ software.

All experimental data were statistically analyzed and visualized using GraphPad Prism 9 software (version 9.0.0, GraphPad Software, San Diego, CA, USA, https://www.graphpad.com/). The results are expressed as mean ± standard deviation. Comparisons between groups were performed using one-way analysis of variance (ANOVA) followed by Tukey’s multiple comparison test. A p-value < 0.05 was considered statistically significant.

## Results

### Targets of components of Eucommiae cortex and their networks

From the database, 147 components of Eucommiae cortex were identified (S1 Table). Applying the screening criteria of OB > 30%, DL > 0.18, and MW < 500, 23 potential active components that may have good efficacy were selected (Table 1). Among them, CID21582571 does not have a corresponding CAS number, so its CID number is provided. GQNBCFQSSDOEHP-LNFBDUAVSA-N lacks both a CAS number and CID number, so its InChIKey is provided.

**Table 1:** 23 potential active components that may have good efficacy.

CAS/CID/Ichkey	Molecule Name	MW	OB (%)	DL
472-15-1	Mairin	456.78	55.37	0.77
573-44-4	liriodendrin_qt	450.48	53.13	0.79
13060-14-5	Yangambin	446.54	57.52	0.8
83-46-5	beta-sitosterol	414.79	36.91	0.75
4449-51-8	Cyclopamine	411.69	55.42	0.82
40957-99-1	(+)-medioresinol	388.45	87.18	0.61
526-06-7	(+)-Eudesmin	386.48	33.28	0.62
2955-23-9	olivil	376.44	62.22	0.4
81426-17-7	AIDS214634	374.42	92.42	0.54
CID21582571	(E)-3-[4-[(1R,2R)-2-hydroxy-2-(4-hydroxy-3-methoxy-phenyl)-1-methylol-ethoxy]-3-methoxy-phenyl]acrolein	374.42	56.31	0.36
GQNBCFQSSDOEHP-LNFBDUAVSA-N	Dehydrodiconiferyl alcohol 4,gamma’-di-O-beta-D-glucopyanoside_qt	358.42	51.44	0.39
4263-87-0	4-[(2S,3R)-5-[(E)-3-hydroxyprop-1-enyl]-7-methoxy-3-methylol-2,3-dihydrobenzofuran-2-yl]-2-methoxy-phenol	358.42	50.75	0.39
2134-98-7	(-)-Tabernemontanine	354.49	58.66	0.6
4423-37-4	Syringetin	346.31	36.82	0.37
32221-58-2	hirsutin_qt	345.35	49.81	0.37
4433-08-3	Dehydrodieugenol	326.42	30.1	0.23
572-59-8	Cinchonan-9-al, 6’-methoxy-, (9R)-	324.46	68.21	0.4
117-39-5	quercetin	302.25	46.43	0.27
466-77-3	Erythraline	297.38	49.17	0.55
83145-47-5	3-beta-Hydroxymethyllenetanshiquinone	294.32	32.16	0.4
35323-91-2	ent-Epicatechin	290.29	48.95	0.24
520-18-3	kaempferol	286.25	41.88	0.24
6754-13-8	Helenalin	262.33	77.01	0.19

MW, Molecular Weight; OB, Oral Bioavailability; DL, Drug-likeness

**Fig 1:**
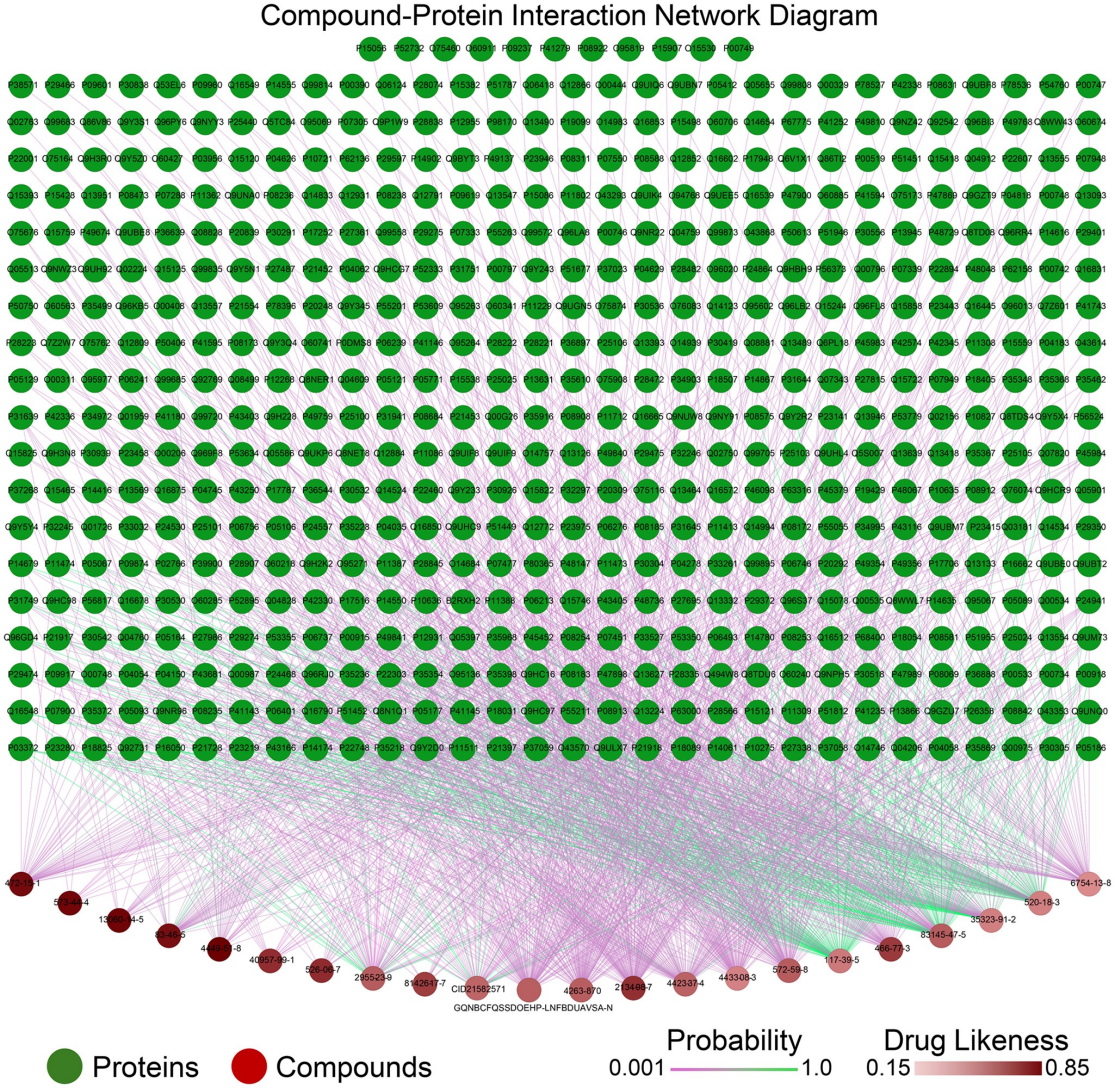
The interaction network diagram of 22 compounds and 581 targets. Green nodes represent proteins, and red nodes represent compounds. The edges connecting these nodes indicate predicted interactions between specific compounds and proteins, with the color of the edges reflecting the probability of interaction; green edges indicate higher probability, while purple edges indicate lower probability. The intensity of the red color in the compound nodes represents the drug-likeness score, with darker red indicating higher drug-likeness.

Among the 23 potential active components selected, two (GQNBCFQSSDOEHP-LNFBDUAVSA-N and 32221-58-2) did not have their SMILES in PubChem. We used their structure files (.mol2) and converted them to SMILES using the Open Babel open-source tool (S1 File). Through the SWISS and TargetNet, we identified 627 potential targets for the potential active components of Eucommiae cortex using their SMILES. When uploaded to the DAVID database, it displayed 581 human targets and 46 targets from other species, resulting in 581 final targets. 22 out of these 23 compounds formed 1,513 connections with the 581 targets. (Fig 1, S2 Table). Among these, 288 connections had a binding probability greater than 0.5, shown as greenish lines in the figure, while connections with a probability less than 0.5 were shown in purplish colors. The nodes corresponding to compounds with higher DL are depicted in darker red, while those with lower DL are shown in lighter red.

### Shared targets of HF, MI, and potential active components, and PPI network

**Fig 2:**
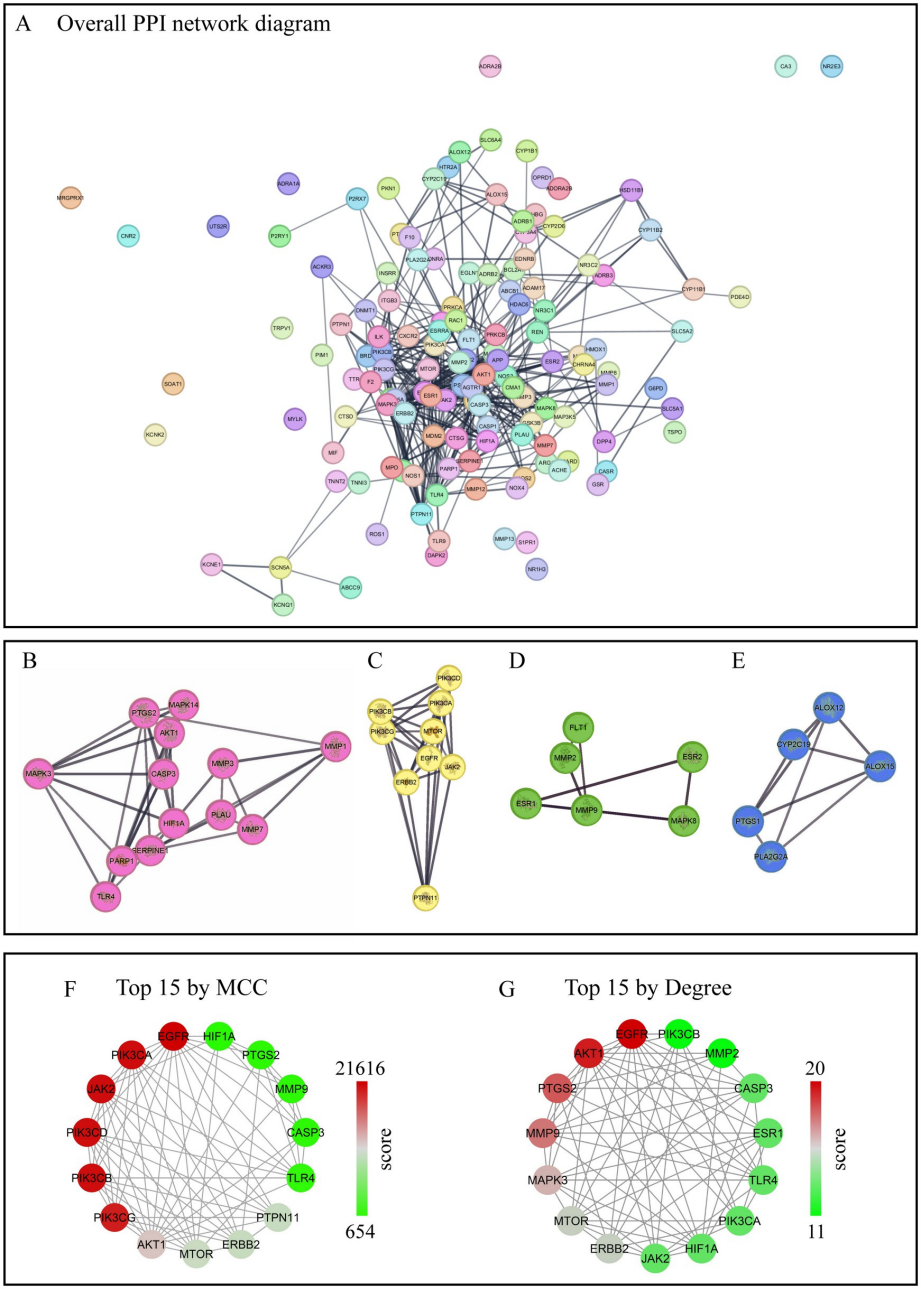
The PPI network diagram of 136 shared proteins. (**A**), The overall PPI network diagram of 136 shared proteins. (**B**)-(**E**), The four core clusters identified using the Molecular Complex Detection plugin in Cytoscape, shown individually. (**F**), The PPI network of the top 15 proteins by MCC. These proteins are part of highly interconnected subnetworks (cliques) within the PPI network. (**G**), The PPI network of the top 15 proteins by Degree. These proteins are key hubs in the PPI network. As in (**F** and **G**), nodes are colored based on their Degree or MCC scores, with red indicating more higher scores. In all panels (**A**)-(**G**), targets are labeled with their name abbreviations. PPI, Protein-Protein Interaction; MCC, Maximal Clique Centrality.

We identified 136 shared targets (S3 Table). A PPI network was generated using these 136 shared targets (Fig 2A). Using the Molecular Complex Detection plugin in Cytoscape, four core clusters (Fig 2B–2E) were identified, comprising a total of 34 targets that cover multiple key biological processes. Core cluster 1 (Fig 2B) is primarily associated with matrix metalloproteinases (MMPs), estrogen receptors (ESR), and MAP kinase signaling pathways. Core cluster 2 (Fig 2C) is related to lipoxygenase (ALOX) and cyclooxygenase (COX) pathways. Core cluster 3 (Fig 2D) involves apoptosis, inflammatory response, and hypoxia response pathways, such as the AKT, mitogen-activated protein kinase (MAPK), and hypoxia-inducible factor 1 (HIF1) signaling pathways. Core cluster 4 (Fig 2E) focuses on the PI3K/AKT/mTOR signaling pathway. We further analyzed these 34 targets using CytoHubba and identified the top 15 proteins with the highest MCC score (Fig 2F) and the top 15 proteins with the highest Degree score (Fig 2G), with 12 proteins (S4 Table) being common between these metrics. These metrics help highlight proteins that may play crucial roles in the biological processes under study. A detailed comparison between the MCC and Degree results shows that despite their different calculation methods, the overlap of 12 common proteins underscores their potential significance as key regulators within the network.

### KEGG and GO enrichment analysis of shared targets of disease and Eucommiae cortex components

The 136 shared targets identified in section “shared targets of HF, MI, and potential active components, and PPI network” were imported into the DAVID database for KEGG and GO enrichment analysis. We obtained 98 KEGG pathways, 70 GOBP terms, 17 GOCC terms, and 32 GOMF terms (S5 Table). These data represent an overview of the potential functions that the potential active components of Eucommiae cortex could achieve through MI and HF-related target proteins.

**Fig 3:**
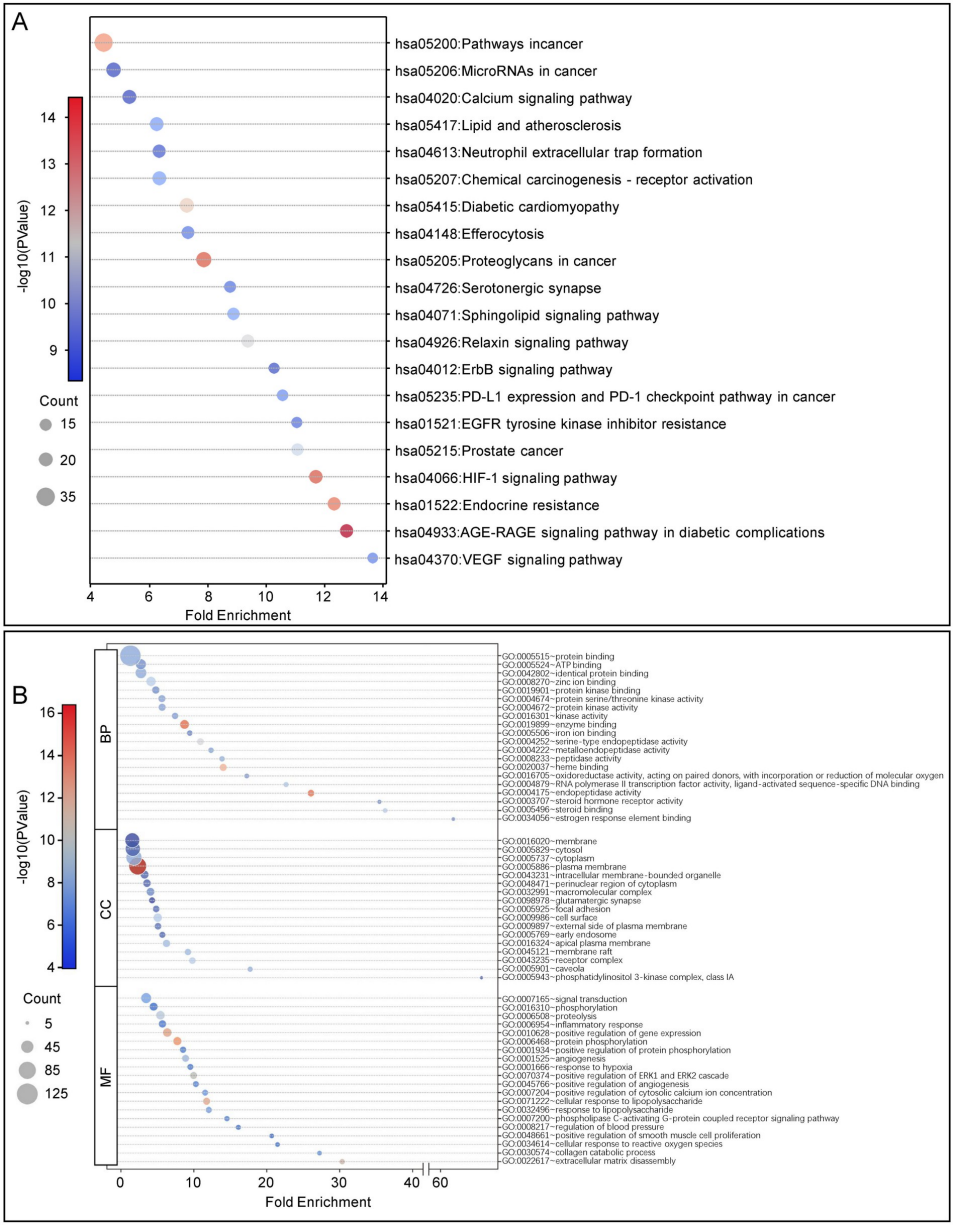
The KEGG and GO enrichment analysis of 136 shared proteins. (**A**) top 20 KEGG enriched based on p-value. The x-axis represents the fold enrichment, which indicates the degree of enrichment for each pathway, and the y-axis lists the names of the pathways. The size of the dots corresponds to the "count," which is the number of proteins associated with each pathway, and the color gradient represents the -log10(p-value), indicating the significance of the enrichment. (**B**) top 20 GO enriched based on p-value. The x-axis represents the fold enrichment, indicating the degree of enrichment for each GO term, and the y-axis lists the specific GO terms categorized into Biological Process (BP), Cellular Component (CC), and Molecular Function (MF). The size of the dots corresponds to the "count," which is the number of proteins associated with each GO term, and the color gradient represents the -log10(p-value), showing the significance of the enrichment. KEGG, Kyoto Ency-clopedia of Genes and Genomes; GO, Gene Ontology; BP, Biological Process; CC, Cellular Component; MF, Molecular Function.

As shown in Fig 3A, the KEGG pathways mainly involve processes such as cancer signaling, microRNA regulation, calcium signaling, lipid metabolism and atherosclerosis, neutrophil extracellular trap formation, angiogenesis, inflammation, and oxidative stress. Among these, pathways such as calcium signaling, lipid metabolism and atherosclerosis, hypoxia-inducible factor 1 (HIF-1) signaling, and vascular endothelial growth factor signaling are significantly associated with cardiovascular diseases. These pathways play crucial roles in myocardial contraction, vascular tone regulation, inflammatory response, oxidative stress, and angiogenesis, which are key mechanisms in the occurrence and development of cardiovascular diseases.

As shown in Fig 3B, the GO biological process enrichment analysis primarily involves processes such as protein phosphorylation, adenosine triphosphate binding, the formation and function of protein complexes, metal ion binding, and redox reactions. Pathways significantly related to cardiovascular diseases include the response to lipopolysaccharide, inflammatory response, angiogenesis, and response to hypoxia. These pathways play important roles in regulating vascular function, immune response, and cell survival [29–31]. In the GO cellular component enrichment analysis, the main cellular structures involved are the cell membrane, cytoplasm, extracellular matrix, and signaling complex. These structures are crucial in intercellular signal transduction, substance transport, and the maintenance of the intra- and extracellular environment. Pathways related to cardiovascular diseases mainly include the early endosome, perinuclear region, and extracellular matrix composition, which affect cellular functions and the stability of tissue structures [32, 33]. In the GO molecular function enrichment analysis, the primary functions involved are protein binding, enzyme binding, metal ion binding, and oxidoreductase activity. These molecular functions play significant roles in protein-protein interactions, enzyme regulation, and the maintenance of cellular redox states. Pathways significantly related to cardiovascular diseases include protein phosphorylation, enzyme binding, and redox reactions, influencing cellular signal transduction and metabolic processes [34, 35].

Through the above analyses, it can be preliminarily confirmed that the effective components of Eucommia exert effects related to oxidative stress and inflammatory responses associated with MI and HF.

### Key proteins highly expressed in myocardium and key KEGG pathways and key GO terms

Key proteins with enriched expression in the myocardium were identified by screening using the Genomics Institute of the Novartis Research Foundation and the Human Protein Atlas. Each source identified six proteins (S6 Table) from the set of 12 key proteins identified in the results of section “shared targets of HF, MI, and potential active components, and PPI network”. By intersecting the two sets, five proteins with enriched expression in the myocardium were obtained (Table 2).

**Table 2:** 5 targets highly expressed in myocardial tissue.

UNIPROT ID	GENE NAME
P31749	AKT serine/threonine kinase 1(AKT1)
O60674	Janus kinase 2(JAK2)
P04626	erb-b2 receptor tyrosine kinase 2(ERBB2)
P42345	mechanistic target of rapamycin kinase(MTOR)
P42336	phosphatidylinositol-4,5-bisphosphate 3-kinase catalytic subunit alpha(PIK3CA)

These 5 protein targets are the highest expressed in myocardial tissue among the 12 key proteins.

**Fig 4:**
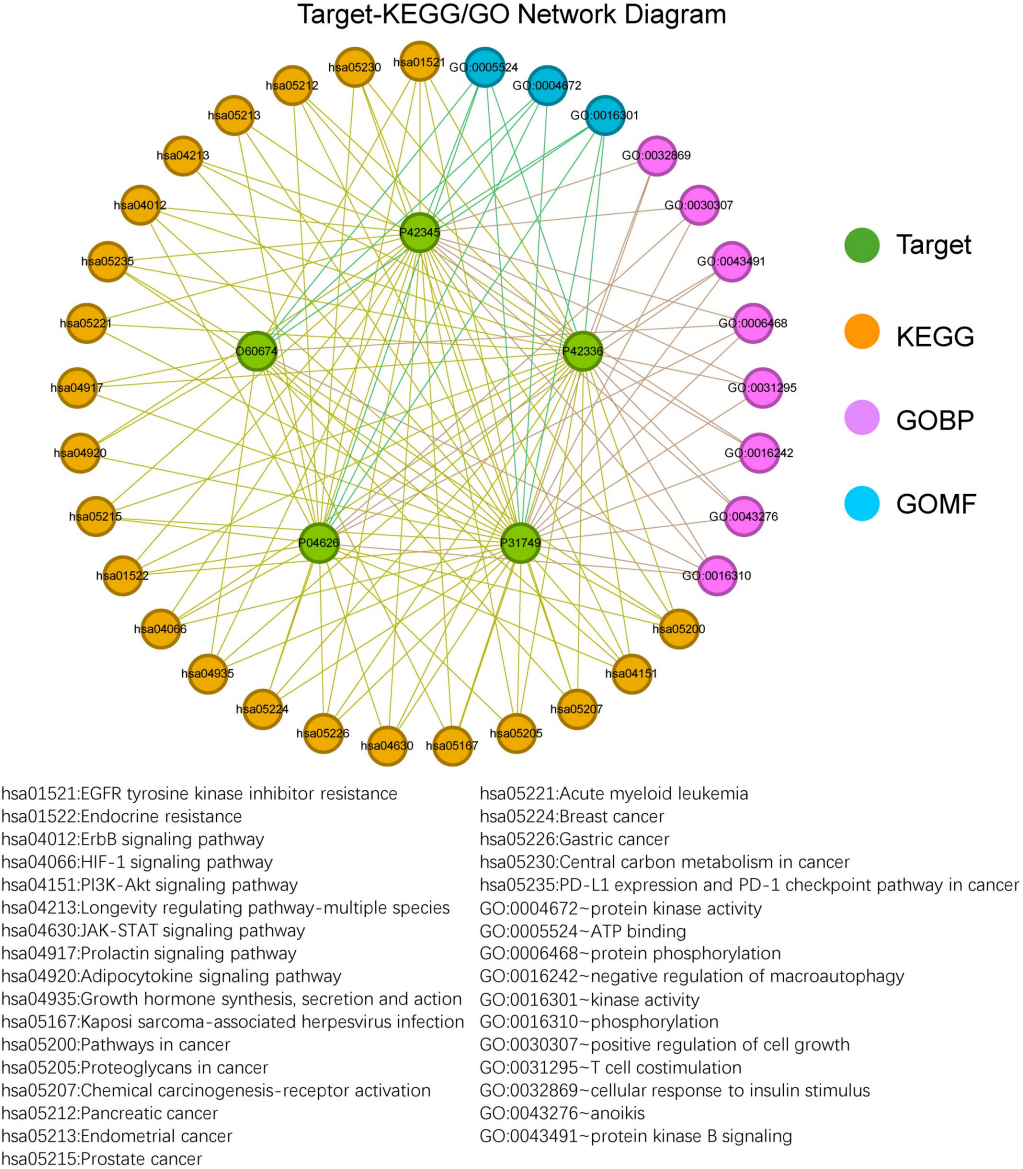
The KEGG and GO enrichment analysis of 5 highly expressed targets in myocardial tissue. Labels in the diagram are represented by KEGG and GO identifiers, as well as the UniProt IDs of target proteins. KEGG, Kyoto Encyclopedia of Genes and Genomes; GOBP, Gene Ontology Biological Process; GOMF, Gene Ontology Molecular Function.

Using the five critical proteins identified in the myocardium in Table 2, KEGG and GO enrichment analyses were performed. This resulted in the identification of 22 KEGG pathways, 8 GOBP, 0 GOCC, and 3 GOMF (S7 Table). The connections between the five proteins and these pathways were visualized (Fig 4). The 22 identified KEGG pathways encompass various biological processes, including cancer-related signaling, metabolic regulation, cell survival and proliferation, and immune responses. Among these pathways, the PI3K/Akt signaling pathway, HIF-1 signaling pathway, Janus kinase/signal transducer and activator of transcription (JAK-STAT) signaling pathway, and Prolactin signaling pathway are known to be closely related to MI and HF and are thus highlighted as key pathways of interest [36–38]. The identified GO terms cover important biological processes and molecular functions such as phosphorylation, apoptosis, autophagy regulation, T cell co-stimulation, cell growth regulation, and insulin response. These processes and functions are closely related to cardiovascular diseases, particularly MI and HF. For example, phosphorylation and protein kinase activity play crucial roles in the regulation of cardiomyocyte function [39] and signal transduction; anoikis and autophagy regulation affect the survival of cardiomyocytes [40, 41].

### Overall network of Eucommiae cortex in treating MI and HF

**Fig 5:**
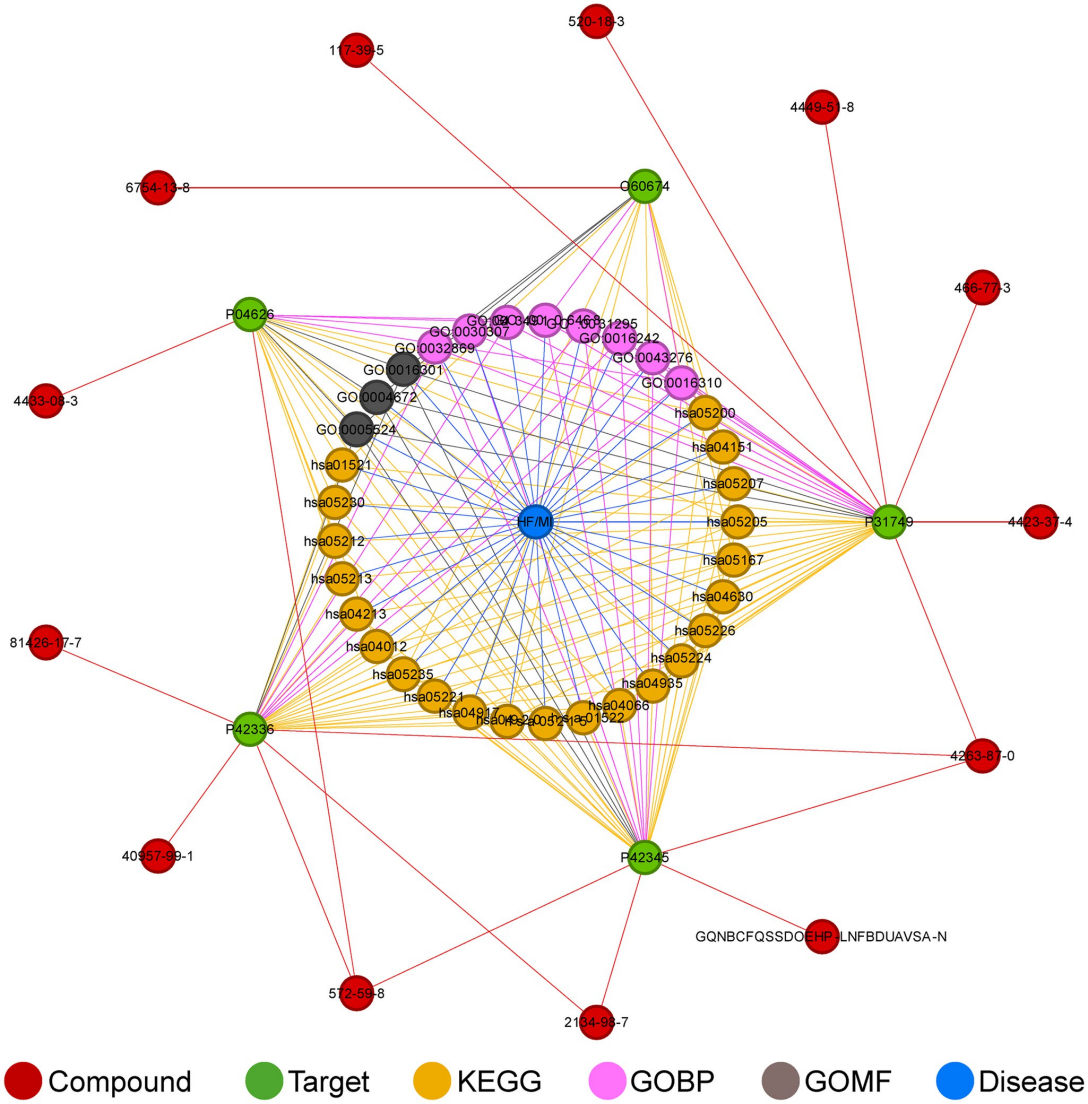
The overall network diagram of myocardial highly expressed targets mediating the effects of Eucommia’s potential active components on KEGG/GO and disease. Labels in the diagram are represented by KEGG and GO identifiers, UniProt IDs of target proteins, and CAS numbers or Ichkey of compounds. HF, heart failure; MI, myocardial infarction; KEGG, Kyoto Encyclopedia of Genes and Genomes; GOBP, Gene Ontology Biological Process; GOMF, Gene Ontology Molecular Function.

The final key proteins, key KEGG pathways, GO terms, and corresponding potential active components of Eucommia were matched and combined with the diseases to create a compound-target-KEGG/GO-disease network diagram (Fig 5). Ultimately, 13 compounds from Eucommia were found to act on 5 key targets, influencing MI and HF through 22 major KEGG pathways and 11 major GO terms (including 8 GOBP, 0GOCC, and 3 GOMF).

### Key PI3K/AKT/mTOR targets in Eucommia cortex components revealed by molecular docking

**Fig 6:**
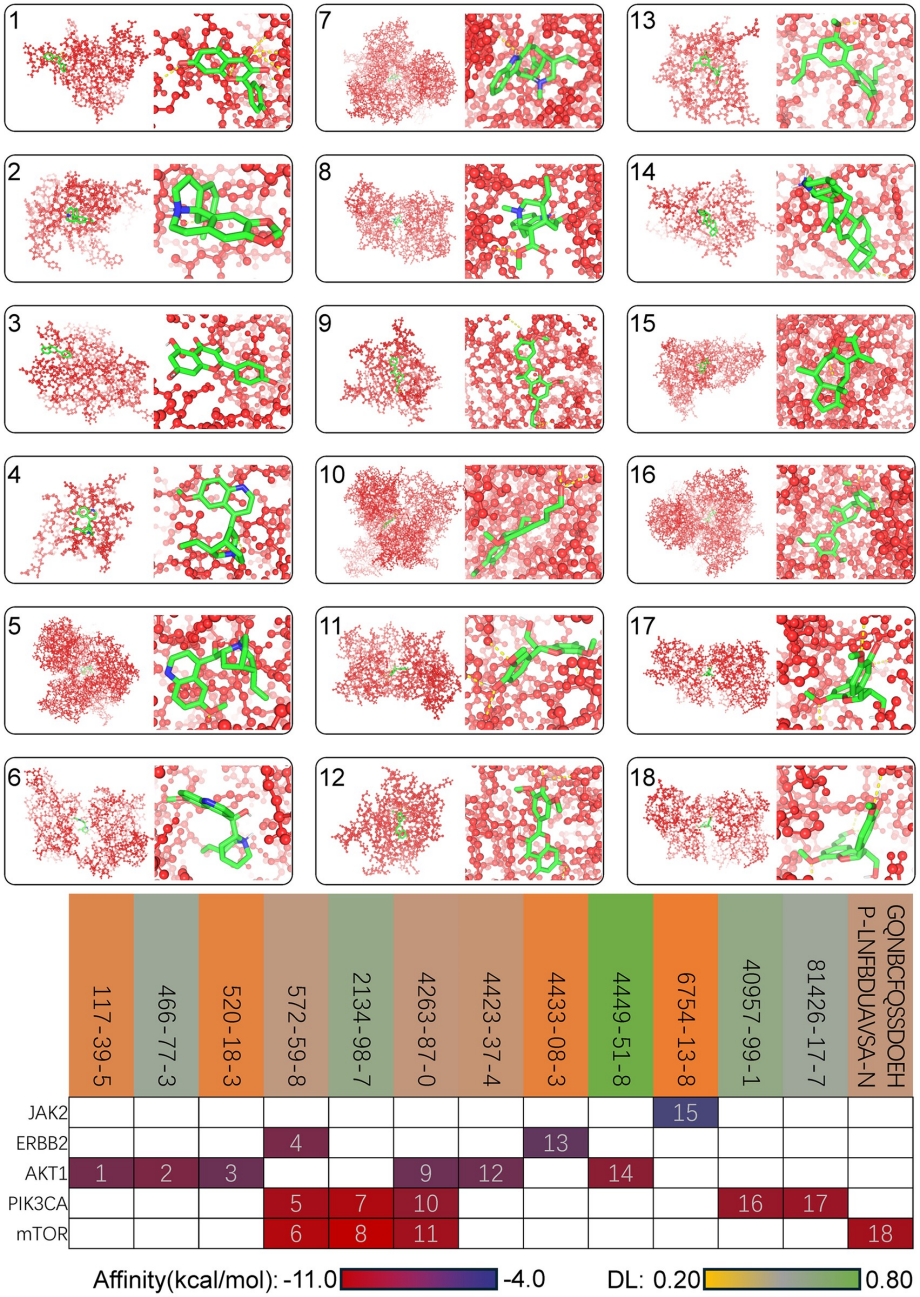
Molecular docking diagrams of 18 types of interactions among compound targets and the heatmap of the lowest binding energy for each interaction. The numbers in the top left corner of the molecular docking diagrams correspond to the numbers in the heatmap. Compounds are labeled with their CAS numbers or Ichkey, and proteins are labeled with gene name abbreviations. DL, drug likeness.

We selected 13 effective components of Eucommia and 5 target proteins in the entire network, performing molecular docking on their 18 connections to evaluate their binding stability. As shown in Fig 6, the binding modes with the lowest binding energy for the 18 dockings are presented. The heatmap shows the variations in the lowest binding energies, with the highest being -4.2 kcal/mol for the binding of 6754-13-8 with O60674, and the lowest being -10.8 kcal/mol for the binding of 2134-98-7 with P42345. Compounds with a greener background color have higher drug-likeness, whereas those with a less green background have lower drug-likeness. The figure shows that in the molecular dockings of the compounds with higher DL values (numbered 2, 7, 8, 14, 16, and 17), they all bind with PI3K, AKT, and mTOR. This indicates that the three key proteins in the PI3K/AKT/mTOR pathway are targets for the effective components of Eucommia. We aim to choose the most upstream PI3K as the target, and further validate through cell experiments whether the effective components of Eucommia can activate downstream AKT/mTOR by regulating PI3K, thereby improving MI and HF. In selecting the components, we will choose those that only act on PI3K and have higher DL values. This points to the compound with CAS number 40957-99-1, commonly known as medioresinol.

### MDRN alleviates OGD-induced damage via PI3K/AKT/mTOR pathway

After incubating cells with various concentrations of MDRN for 24 hours, we observed significant cell death at concentrations greater than 120 μM (S1 Fig). Therefore, we selected 120 μM as the final intervention concentration. A concentration of 60 μM was used as the low-dose intervention group.

**Fig 7:**
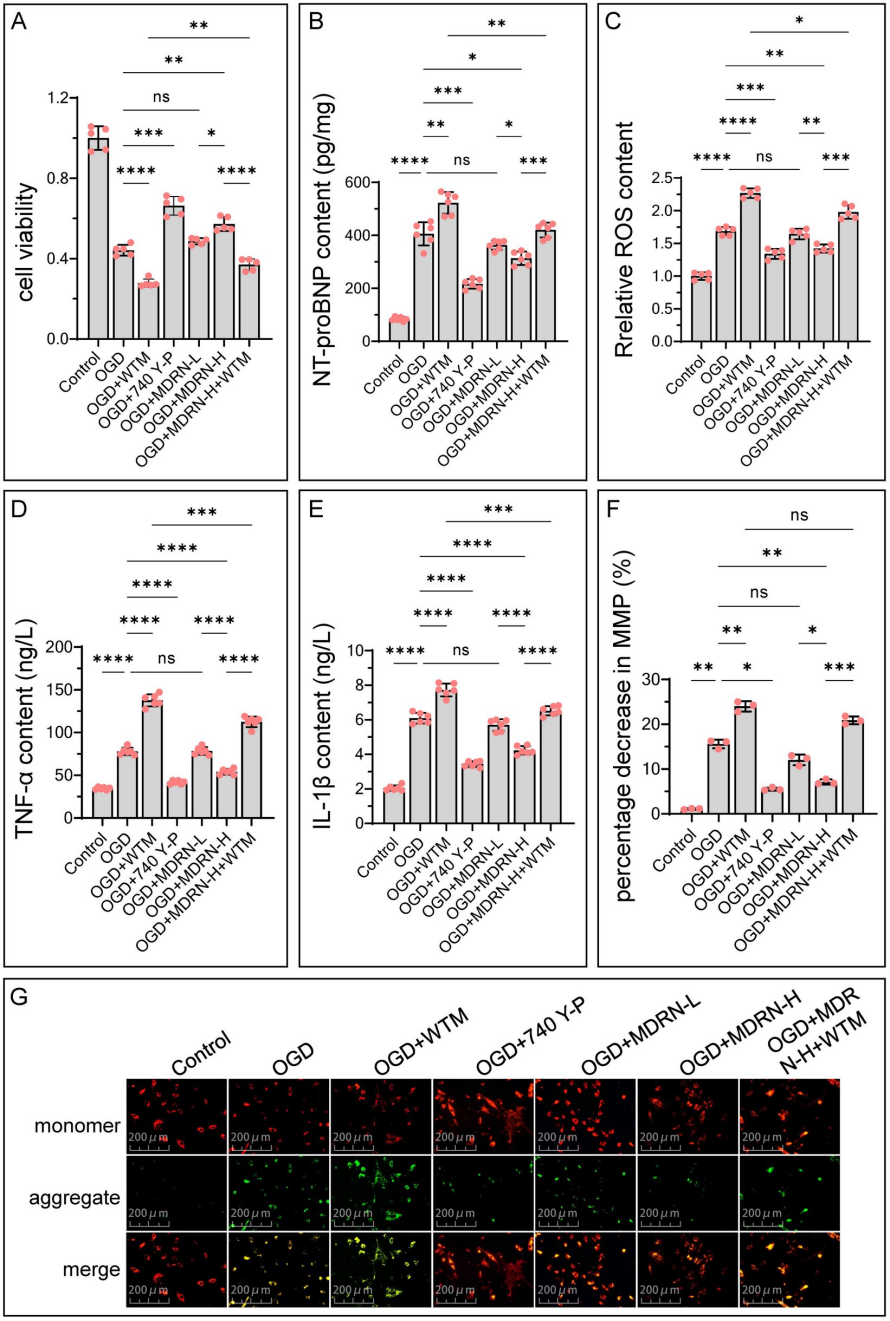
The conditions of heart failure, oxidative stress, inflammatory response, and mito-chondrial membrane potential in each group of cells. (**G**) Displays images under a fluorescence microscope after JC-1 staining. Each (red circle) represents a sample data point. ns p≥0.05, *p<0.05, **p<0.01, ***p<0.001, ****p<0.0001. NT-proBNP, N-terminal pro b-type Natriuretic Peptide; ROS, Reactive Oxygen Species; TNF-α, Tumor Necrosis Factor-alpha; IL-1β, Interleukin-1 beta; MMP, Mitochondrial Membrane Potential; OGD, Oxygen and Glucose Deprivation; WTM, Wortmannin; MDRN, Medioresinol; L, Low Dose; H, High Dose.

As shown in Fig 7A and 7B, the OGD model cells exhibited reduced viability and elevated NT-proBNP levels, indicating successful construction of a HF induced by OGD model. Inhibiting PI3Kα with WTM exacerbated these effects, whereas the PI3Kα agonist 740 Y-P improved them. Similarly, MDRN was able to counteract OGD-induced damage. The protective effect of MDRN was attenuated when PI3K was inhibited with WTM. However, cell viability remained higher than in the OGD+WTM group, and NT-proBNP levels were lower than in the OGD+WTM group. This suggests that PI3K is an effective target for MDRN in improving post-MI HF, though not the sole target. Furthermore, this effect is concentration-dependent, with suboptimal effects observed at lower concentrations of MDRN.

Similarly, in oxidative stress (Fig 7C) and inflammatory responses (Fig 7D and 7E), the OGD model showed more severe conditions compared to the control group, characterized by significantly elevated levels of ROS, TNF-α, and IL-1β. Following MDRN treatment, ROS, TNF-α, and IL-1β levels were markedly reduced. However, the therapeutic effect of MDRN was diminished when PI3Kα was inhibited. Cardiomyocytes contain abundant mitochondria, and their function is closely related to mitochondrial activity, reflected by mitochondrial membrane potential, which can indicate oxidative stress levels effectively. As shown in Fig 7F and 7G, JC-1 mitochondrial staining fluorescence images demonstrated that OGD caused a decrease in mitochondrial membrane potential. Specific inhibition of PI3Kα further reduced mitochondrial membrane potential, while activation restored it. MDRN, similar to a PI3K activator, effectively restored mitochondrial membrane potential, which was attenuated by PI3Kα inhibitors.

**Fig 8:**
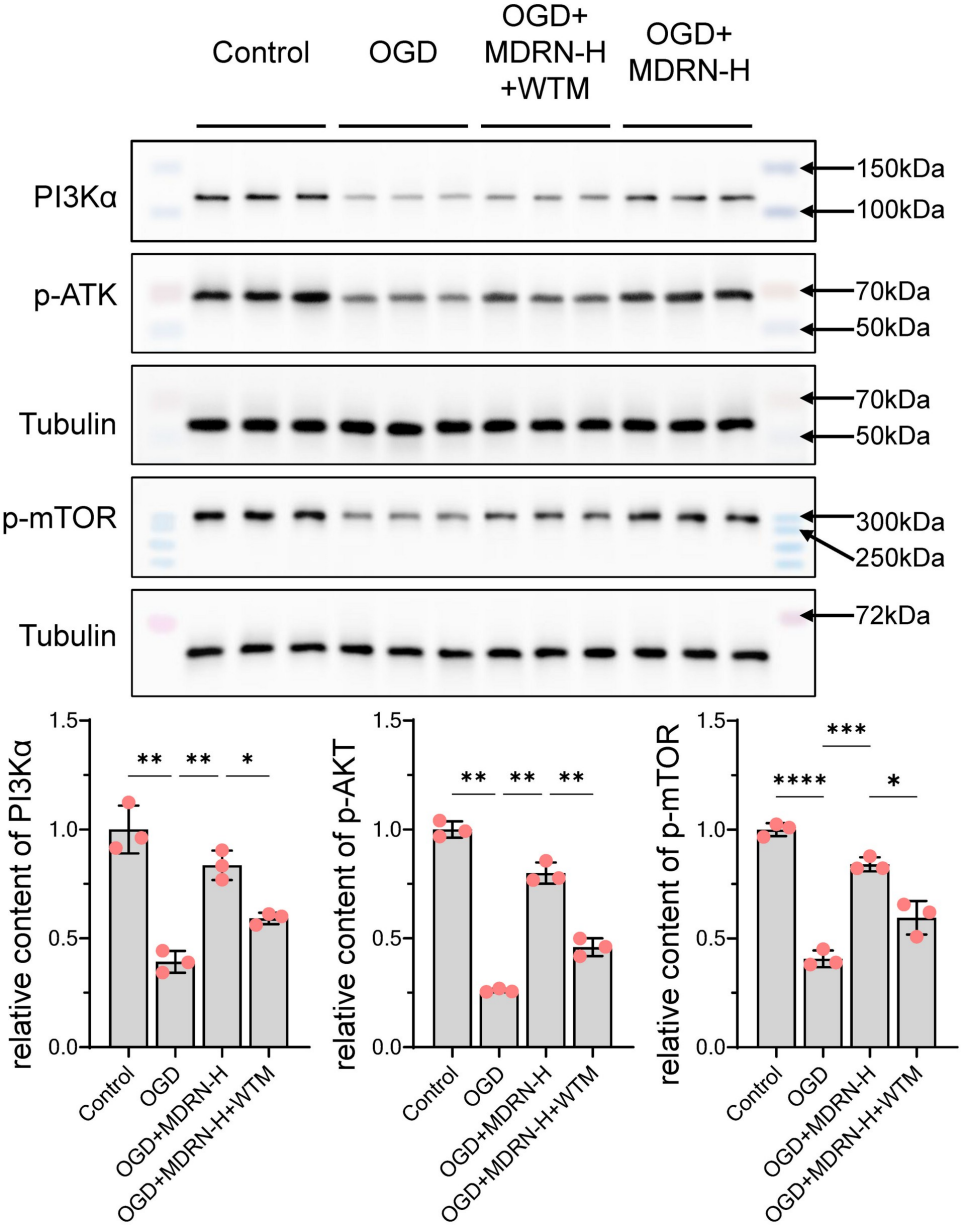
The Western blot detection results of PI3K, p-AKT, and p-mTOR. Each (red circle) represents a sample data point. ns p≥0.05, *p<0.05, **p<0.01, ***p<0.001, ****p<0.0001. OGD, Oxygen and Glucose Deprivation; WTM, Wortman-nin; MDRN-H, High Dose Medioresinol; PI3Kα, Phosphoinositide 3-kinase alpha; p-AKT, Phosphorylated Protein Kinase B; p-mTOR, Phosphorylated Mechanistic Target of Rapamycin.

Oxidative stress and inflammatory responses are regulatory targets of the PI3Kα-AKT-mTOR pathway. As shown in Fig 8, compared to the control group, the OGD group exhibited varying degrees of reduction in PI3K, p-AKT, and p-mTOR levels. Following MDRN treatment, there was a recovery in PI3K, p-AKT, and p-mTOR levels compared to the OGD group. However, inhibition of PI3K reduced the extent of MDRN-induced recovery of p-AKT and p-mTOR. Therefore, MDRN’s improvement in MI and HF is closely related to the PI3K/AKT/mTOR pathway, with PI3K being a key target for MDRN to activate this pathway.

## Discussion

This study systematically explored the potential therapeutic effects and mechanisms of Eucommiae cortex in the treatment of HF following MI. Utilizing network analysis and molecular docking approaches, we screened and analyzed the potential active compounds of Eucommiae cortex, and validated their anti-inflammatory and antioxidant properties of MDRN through in vitro experiments. Specifically, 23 potential active compounds were identified from the database using stringent criteria (OB ≥ 30%, DL ≥ 0.18, and MW < 500) to ensure high drug potential. Out of the predicted 581 potential targets, 136 targets were found to be associated with HF and MI through PPI. KEGG, and GO enrichment analyses, indicating that Eucommiae cortex may exert its effects through pathways such as PI3K/AKT/mTOR, HIF-1, and JAK-STAT, which are involved in calcium signaling, lipid metabolism, inflammatory response, and oxidative stress. The PI3K/AKT/mTOR, HIF-1, and JAK-STAT pathways play crucial roles in cellular processes relevant to cardiovascular health, particularly in the context of MI and HF. The PI3K/AKT/mTOR pathway is pivotal for cellular growth, proliferation, and survival, and has been shown to enhance cardiac function by promoting angiogenesis, reducing apoptosis, and improving mitochondrial function, thereby mitigating damage from MI and HF [42]. The HIF-1 pathway, activated in response to hypoxia, supports cellular adaptation to low oxygen levels by promoting angiogenesis and metabolic adjustments, which improve tissue survival and function during MI [43, 44]. The JAK-STAT pathway, integral to inflammatory and cellular stress responses, has emerged as a critical target in cardiovascular diseases associated with autoimmune disorders. Research has indicated that JAK-STAT signaling contributes to inflammation and cardiovascular risk, and its inhibitors may offer new treatment strategies [45]. These pathways collectively contribute to essential mechanisms like oxidative stress regulation, lipid metabolism, calcium signaling, and inflammatory responses, which are vital for cardiovascular health.

Molecular docking analysis indicated that the selected compounds could stably bind to their targets, especially PI3K, AKT, and mTOR. We chose MDRN, which acts on the PI3K/Akt/mTOR pathway, for in-depth study and validated its effects on MI-induced HF through in vitro experiments. The results showed that MDRN could significantly improve oxidative stress and inflammatory response in myocardial ischemia-hypoxia model cells by activating the PI3K/AKT/mTOR signaling pathway, enhancing cell viability and mitochondrial membrane potential. This is consistent with previous studies that have also demonstrated that activating the PI3K/AKT/mTOR pathway can reduce cell apoptosis, improve mitochondrial function, and enhance overall cell survival under ischemic conditions [46]. This study not only confirms the role of the PI3K/AKT/mTOR pathway in the cardiovascular protective effects of Eucommiae cortex but also provides specific cellular-level regulatory evidence, offering a theoretical basis for the treatment of MI-induced HF with Eucommiae cortex.

Medioresinol, a lignan compound found in flaxseed and Eucommiae cortex, is known for its antioxidant, anti-inflammatory, and anticancer properties. Current research on MDRN is limited. It has been shown that brain microvascular endothelial cells undergo pyroptosis after ischemic stroke, and MDRN, as a peroxisome proliferator-activated receptor gamma coactivator 1-alpha activator, ameliorates pyroptosis and ischemic brain injury by reducing mitochondrial ROS via the peroxisome proliferator-activated receptor alpha/glutamate oxaloacetate transaminase 1 axis [47]. Another study found that MDRN leads to ROS accumulation in fungi [48]. Our research further supports that MDRN reduces oxidative stress post-ischemia. The anti-inflammatory effects of lignans and their metabolites are well-documented [49], but there are no direct studies on the anti-inflammatory effects of MDRN. Our study is the first to demonstrate through in vitro experiments that MDRN can inhibit ischemia-induced inflammatory responses, reducing TNF-α and IL-1β levels.

However, this study has certain limitations. Firstly, the single compound MDRN cannot fully represent the entire spectrum of Eucommiae cortex’s therapeutic functions. Secondly, due to budget constraints, this research was limited to in vitro experiments. Therefore, we plan to conduct further studies to validate the overall efficacy and mechanisms of Eucommiae cortex in treating MI-induced HF, using both animal models and cell models to explore the therapeutic effects and molecular mechanisms of Eucommiae cortex and its compounds.

## Conclusions

This study utilized network analysis and molecular docking to systematically analyze the potential targets and mechanisms of Eucommiae cortex in MI-induced HF, and validated its anti-inflammatory and antioxidant capabilities through in vitro experiments. The findings suggest that the active compound MDRN may exert therapeutic effects on MI-induced HF by activating the PI3K/AKT/mTOR signaling pathway, inhibiting inflammatory response, and reducing oxidative stress, thus improving mitochondrial membrane potential. These results provide a new theoretical foundation and research direction for the application of Eucommiae cortex in the treatment of cardiovascular diseases. Future research should aim to further promote the application and development of Eucommiae cortex in cardiovascular disease treatment.

## Supporting information

S1 Table 147 components of Eucommia cortex from TCMSP. (XLSX)

S2 Table The 1513 connections formed between the 22 compounds and 581 targets. (XLSX)

S3 Table The 136 shared targets between the disease and compounds and their UniProt IDs. (XLSX)

S4 Table The 12 shared targets identified by both MCC and degree analysis. (XLSX)

S5 Table KEGG and GO enrichment tables for the 136 shared targets between the disease and compounds. (XLSX)

S6 Table Six myocardial highly expressed proteins screened through the GNF and six myocardial highly expressed proteins screened through the HPA. (XLSX)

S7 Table KEGG and GO enrichment tables for the 5 key proteins jointly screened by GNF and HPA. (XLSX)

S1 Fig CCK-8 assay to determine the cell viability of H9c2 cells cultured with different concentrations of MDRN for 24 hours. (PDF)

S1 File Two compounds (GQNBCFQSSDOEHP-LNFBDUAVSA-N and 32221-58-2) and their SMILES obtained through conversion. (ZIP)

S1 Raw images (PDF)
