# Supplementary material for: Medioresinol from Eucommiae cortex improves myocardial infarction-induced heart failure through activation of the PI3K/AKT/mTOR pathway: A network analysis and experimental study
Source: PLoS One. 2024 Sep 27;19(9):e0311143. doi: 10.1371/journal.pone.0311143 (PMC11433142; doi:10.1371/journal.pone.0311143)
Supplement: S1 Fig — (PDF) [file pone.0311143.s008.pdf]

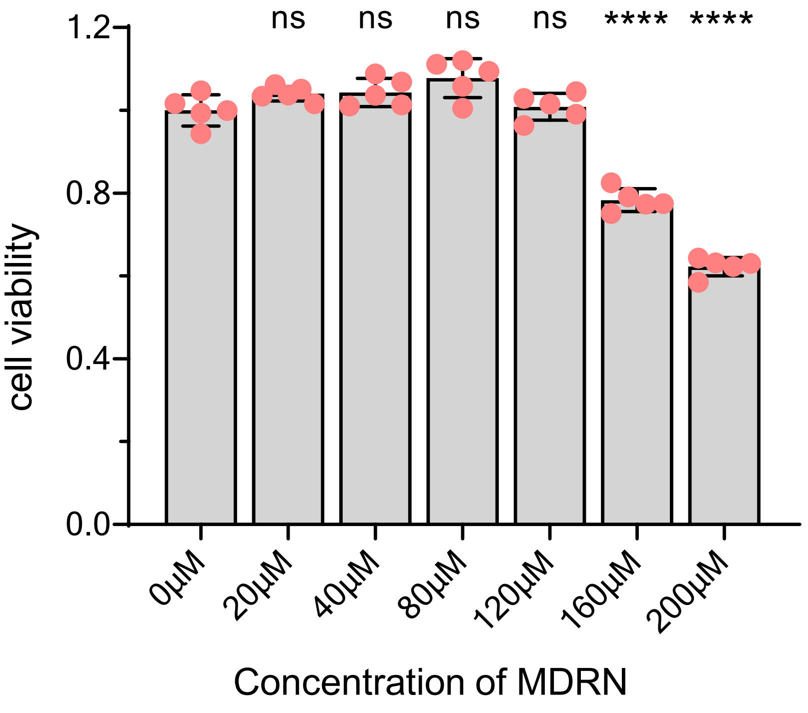

Supplement Figure 1.CCK-8 assay to determine the cell viability of H9c2 cells cultured with different concentrations of MDRN for 24 hours. ns $p \geq 0.05$ vs control group, ****$p < 0.0001$ vs control group. MDRN, Medioresinol.
