# Supplementary material for: Medioresinol from Eucommiae cortex improves myocardial infarction-induced heart failure through activation of the PI3K/AKT/mTOR pathway: A network analysis and experimental study
Source: PLoS One. 2024 Sep 27;19(9):e0311143. doi: 10.1371/journal.pone.0311143 (PMC11433142; doi:10.1371/journal.pone.0311143)
Supplement: S1 Raw images — (PDF) [file pone.0311143.s010.pdf]

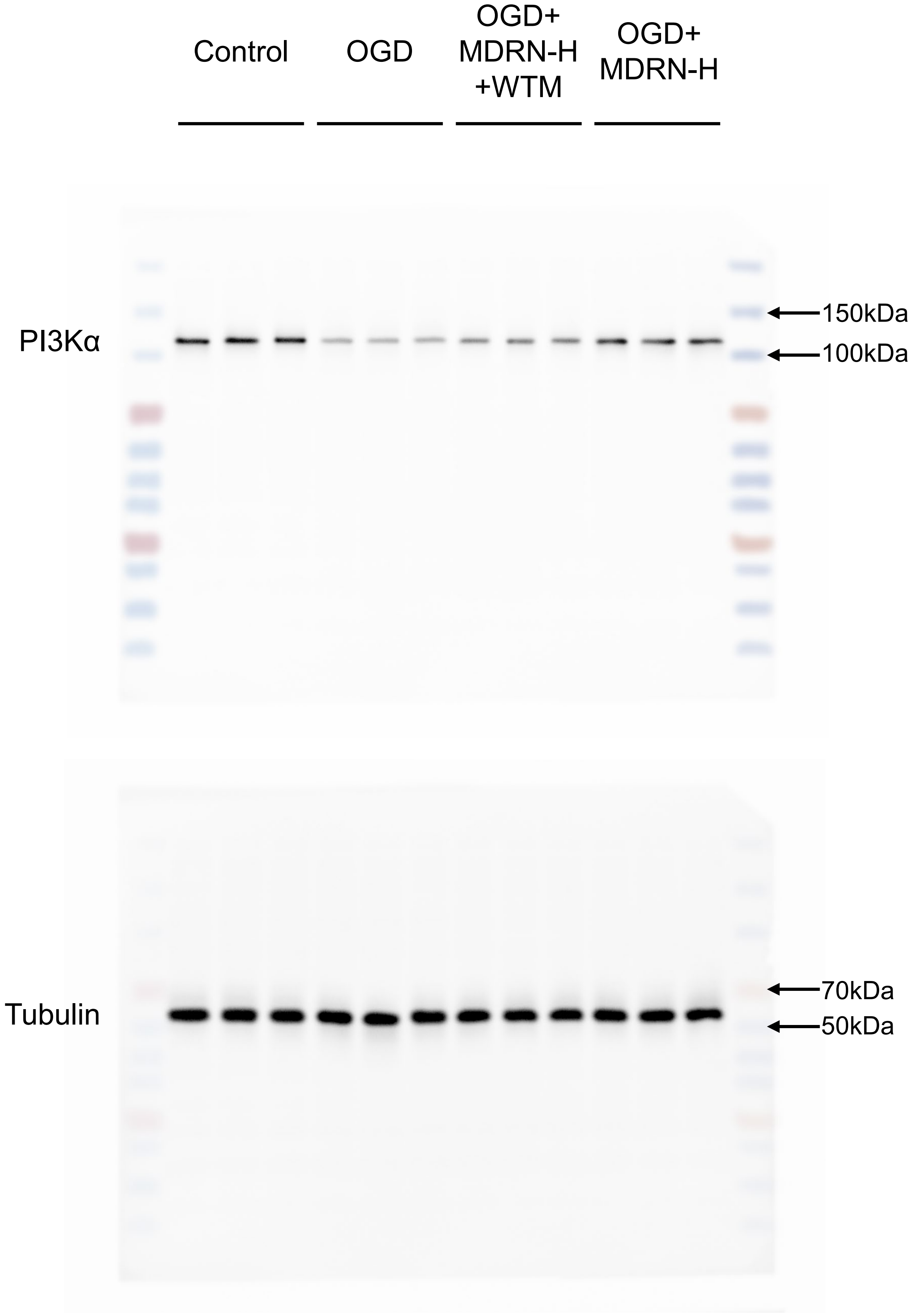

Control
OGD
OGD+ MDRN-H +WTM
OGD+ MDRN-H
PI3Kα
150kDa
100kDa
Tubulin
70kDa
50kDa

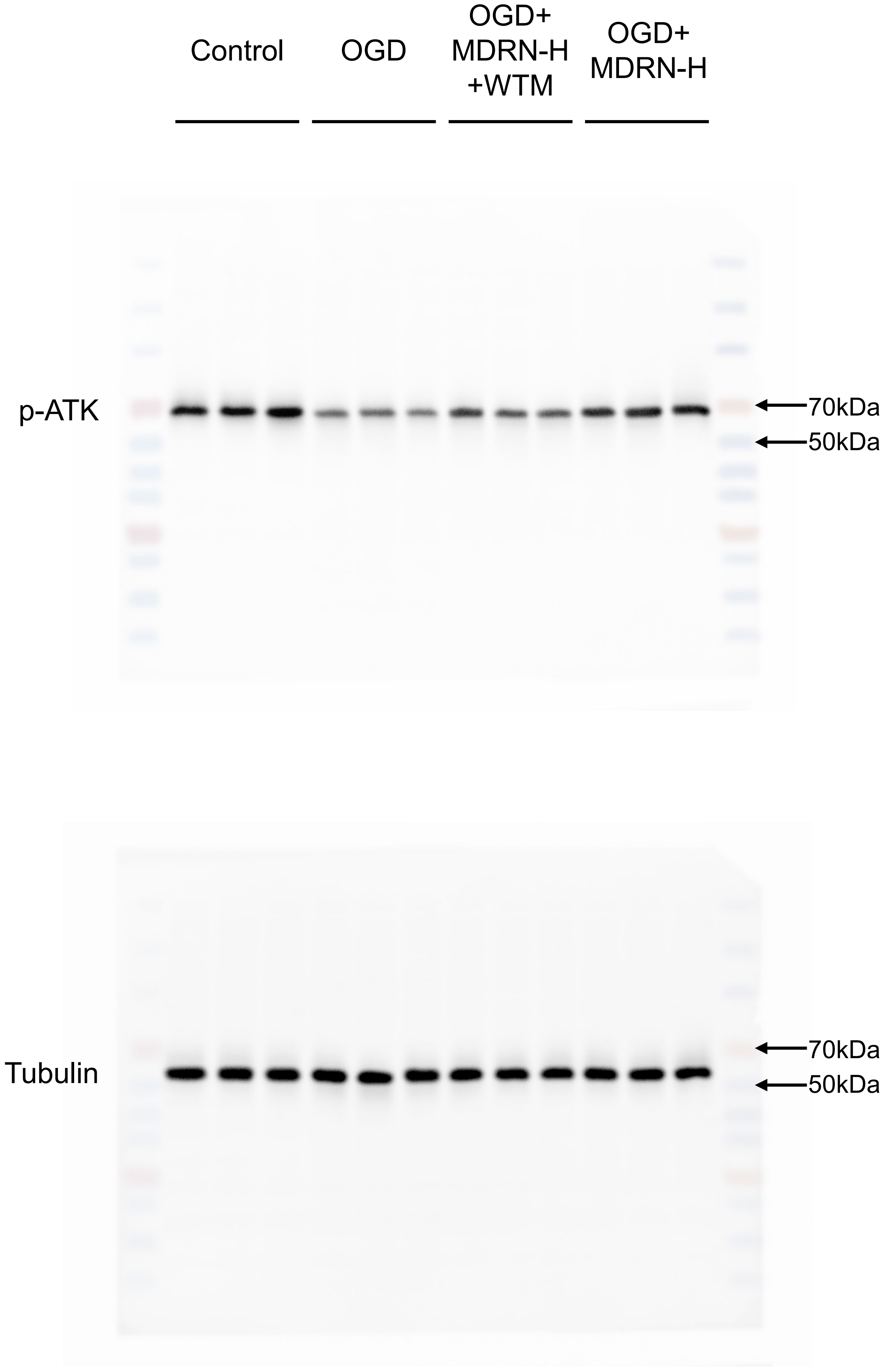
Control
OGD
OGD+
MDRN-H
+WTM
OGD+
MDRN-H
p-ATK
70kDa
50kDa
Tubulin
70kDa
50kDa

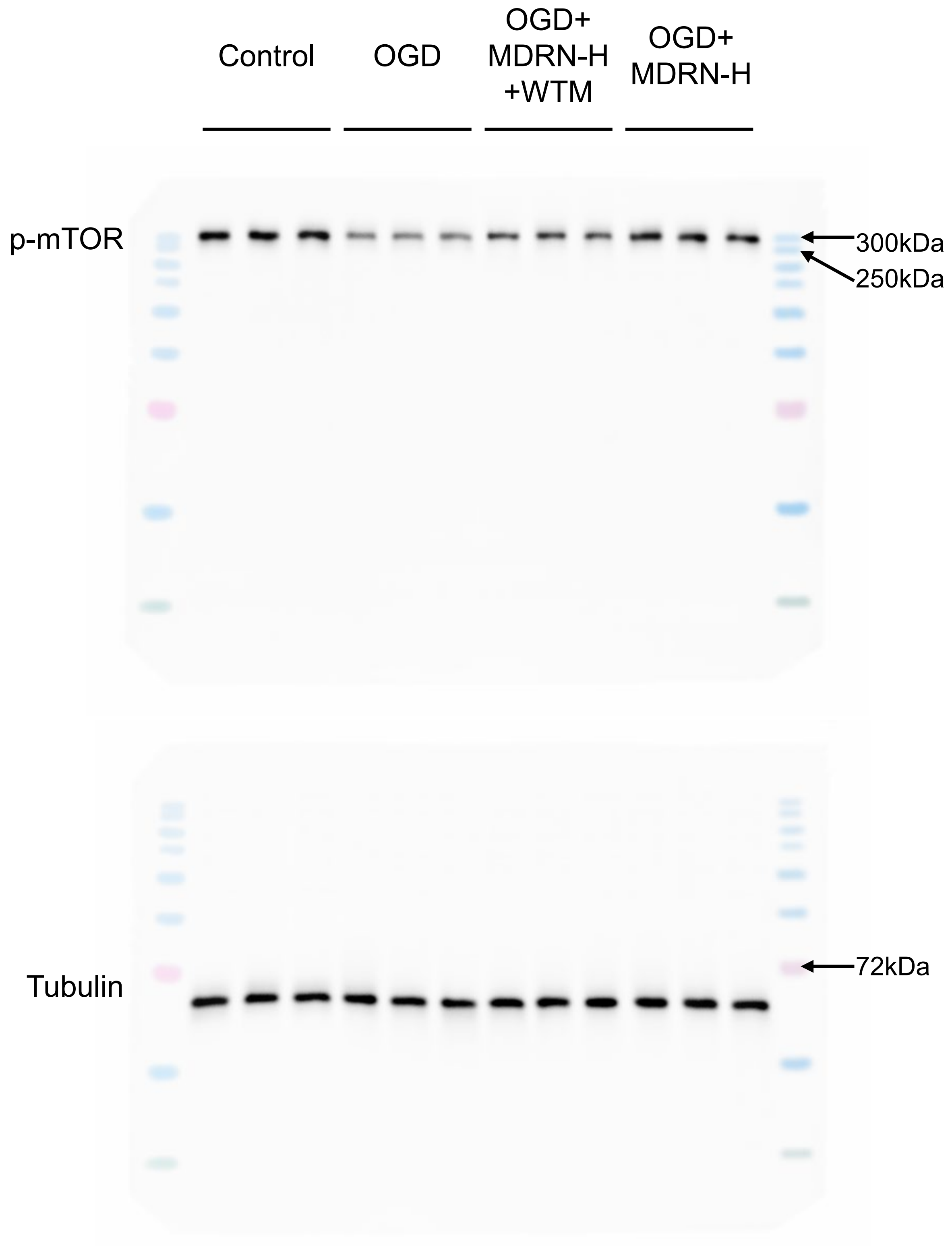
Control
OGD
OGD+
MDRN-H
+WTM
OGD+
MDRN-H
p-mTOR
300kDa
250kDa
Tubulin
72kDa
